# Common Peripheral Immunity Mechanisms in Multiple Sclerosis and Alzheimer's Disease

**DOI:** 10.3389/fimmu.2021.639369

**Published:** 2021-02-19

**Authors:** Barbara Rossi, Bruno Santos-Lima, Eleonora Terrabuio, Elena Zenaro, Gabriela Constantin

**Affiliations:** ^1^Section of General Pathology, Department of Medicine, University of Verona, Verona, Italy; ^2^The Center for Biomedical Computing (CBMC), University of Verona, Verona, Italy

**Keywords:** multiple sclerosis, Alzheimer's disease, neuroinflammation, neutrophils, monocytes, T cells

## Abstract

Neurodegenerative diseases are closely related to inflammatory and autoimmune events, suggesting that the dysregulation of the immune system is a key pathological factor. Both multiple sclerosis (MS) and Alzheimer's disease (AD) are characterized by infiltrating immune cells, activated microglia, astrocyte proliferation, and neuronal damage. Moreover, MS and AD share a common pro-inflammatory signature, characterized by peripheral leukocyte activation and transmigration to the central nervous system (CNS). MS and AD are both characterized by the accumulation of activated neutrophils in the blood, leading to progressive impairment of the blood–brain barrier. Having migrated to the CNS during the early phases of MS and AD, neutrophils promote local inflammation that contributes to pathogenesis and clinical progression. The role of circulating T cells in MS is well-established, whereas the contribution of adaptive immunity to AD pathogenesis and progression is a more recent discovery. Even so, blocking the transmigration of T cells to the CNS can benefit both MS and AD patients, suggesting that common adaptive immunity mechanisms play a detrimental role in each disease. There is also growing evidence that regulatory T cells are beneficial during the initial stages of MS and AD, supporting the link between the modulatory immune compartments and these neurodegenerative disorders. The number of resting regulatory T cells declines in both diseases, indicating a common pathogenic mechanism involving the dysregulation of these cells, although their precise role in the control of neuroinflammation remains unclear. The modulation of leukocyte functions can benefit MS patients, so more insight into the role of peripheral immune cells may reveal new targets for pharmacological intervention in other neuroinflammatory and neurodegenerative diseases, including AD.

## Introduction

Multiple sclerosis (MS) and Alzheimer's disease (AD) are two of the most widely studied central nervous system (CNS) pathologies. MS is the most common inflammatory neurological disease in young adults, whereas AD is a neurodegenerative disorder that occurs more frequently in the elderly population and is the most common type of dementia. The number of MS and AD patients is growing continuously, highlighting the need to find new disease mechanisms and new therapeutic approaches (1, 2). MS and AD are both multifactorial diseases and the identification of their etiopathogenetic mechanisms is challenging. Genetic risk factors and environmental triggers are the principal risk factors for both MS and AD (3, 4).

From the neuropathological perspective, the early phases of MS, defined as relapsing-remitting MS (RRMS), are characterized by primary demyelination areas known as plaques, which are located in the white and gray matter. In contrast, the final phases (secondary progressive MS) are associated with axonopathy, neuronal death, and synaptic loss, correlating with the permanent motor disability classically shown by MS patients (5). AD neuropathology is characterized by two main hallmarks: amyloid β plaques and tau tangles. Both structures are formed from aggregated proteins, in one case due to the incorrect processing of amyloid precursor protein (APP), and in the other due to the hyperphosphorylation of tau, a microtubule-associated protein required to maintain neuronal architecture and function.

The involvement of immune and inflammatory reactions in the pathogenesis of MS has been understood for decades, but the same association was only recently identified in AD. The concept of CNS exclusion from surveillance and inflammatory responses mediated by peripheral immune cells was reconsidered more than 15 years ago describing how circulating immune cells enter into the brain for protective tissue immunosurveillance and has been recently reviewed following the discovery of the cerebral lymphatic system and its role in CNS physiology (6–8). Furthermore, strong evidence of peripheral immune cell trafficking into the CNS has been provided during the immune responses that occur during MS and its animal model, experimental autoimmune encephalomyelitis (EAE), confirming that the CNS is not an immune-privileged environment (9). More recently, immune cell trafficking has also been documented in AD and was shown to be detrimental in transgenic mice with AD-like disease (10, 11). Neuroinflammation in both MS and AD is also characterized by the activation of microglia and astrocytes, leading to the secretion of pro-inflammatory cytokines and chemokines that recruit more immune cells from the periphery to the CNS (12, 13). Interestingly, the specialized pro-resolving lipid mediators, which mediate inflammation resolution and reduce neutrophil and monocytes infiltration into the brain, are impaired in MS and AD patients and their levels correlate with disease severity (14–16). These defects in the resolution pathways further emphasize the common detrimental role of peripheral immune cells in the maintenance of neuroinflammatory process in MS and AD.

Inflammation during neurodegenerative disorders is not restricted to the CNS. Indeed, systemic inflammation has been confirmed in MS and AD, including the secretion of pro-inflammatory cytokines in peripheral domains such as the blood, cerebrospinal fluid (CSF), liver, and gut (12, 17–20). The involvement of peripheral inflammation mechanisms and immune cells in MS and AD provides strong evidence of immune dysregulation, but it is unclear whether this is a causal link in each disease or a secondary phenomenon triggered by brain injury.

Whereas, the role of humoral response has been reviewed elsewhere, here we discuss the common immune mechanisms in MS and AD and describe how neutrophils, monocytes and T cell subpopulations use similar mechanisms in MS and AD to migrate into the CNS and induce neuroinflammation and tissue damage (21). These insights suggest that interfering with shared cellular and molecular mechanisms may lead to common therapeutic approaches for MS and AD.

## Neutrophils: The Emerging Players in MS and AD

Neutrophils are highly reactive leukocytes with a frontline role in the maintenance of tissue homeostasis during pathological conditions, including infections and tissue damage (22). Neutrophils are highly adaptable cells due to their remarkable plasticity, and can therefore adjust their phenotype and functions in response to various environmental stimuli, triggering acute inflammatory responses (23). However, when prolonged tissue stress and damage induce sterile inflammation, neutrophils play a more subtle detrimental role, leading to chronic tissue damage that can promote pathological conditions such as autoimmunity and neurodegenerative diseases if left uncontrolled (24). These heterogeneous cells have attracted significant interest given their ability to facilitate sterile and chronic inflammation (24, 25).

Infiltrating neutrophils have been detected in the brains of MS and AD patients (10, 26). Evidence for the early involvement of neutrophils in MS includes their correlation with hyperacute lesions and altered blood-brain barrier (BBB) permeability in humans, and their involvement in the preclinical phase of EAE and acute relapses in these animal models (26, 27). Studies in AD models also suggest that neutrophils may contribute to the initial disease stages, which are characterized by increased BBB permeability, neutrophil intravascular adhesion, and invasion of the CNS (10, 11). Indeed, in animal models of both MS and AD, neutrophils accumulate in the brain before clinical manifestation, representing a major source of inflammatory mediators during early disease stages (10, 28, 29). We and others have shown that blocking neutrophil recruitment at early disease stages reduces the disease burden and tissue damage in animal models of both MS and AD (10, 30, 31). However, neutrophils continue to accumulate in the CNS throughout the disease course, suggesting these cells also play a role in disease progression and chronicity (10, 28, 29).

**Figure 1:**
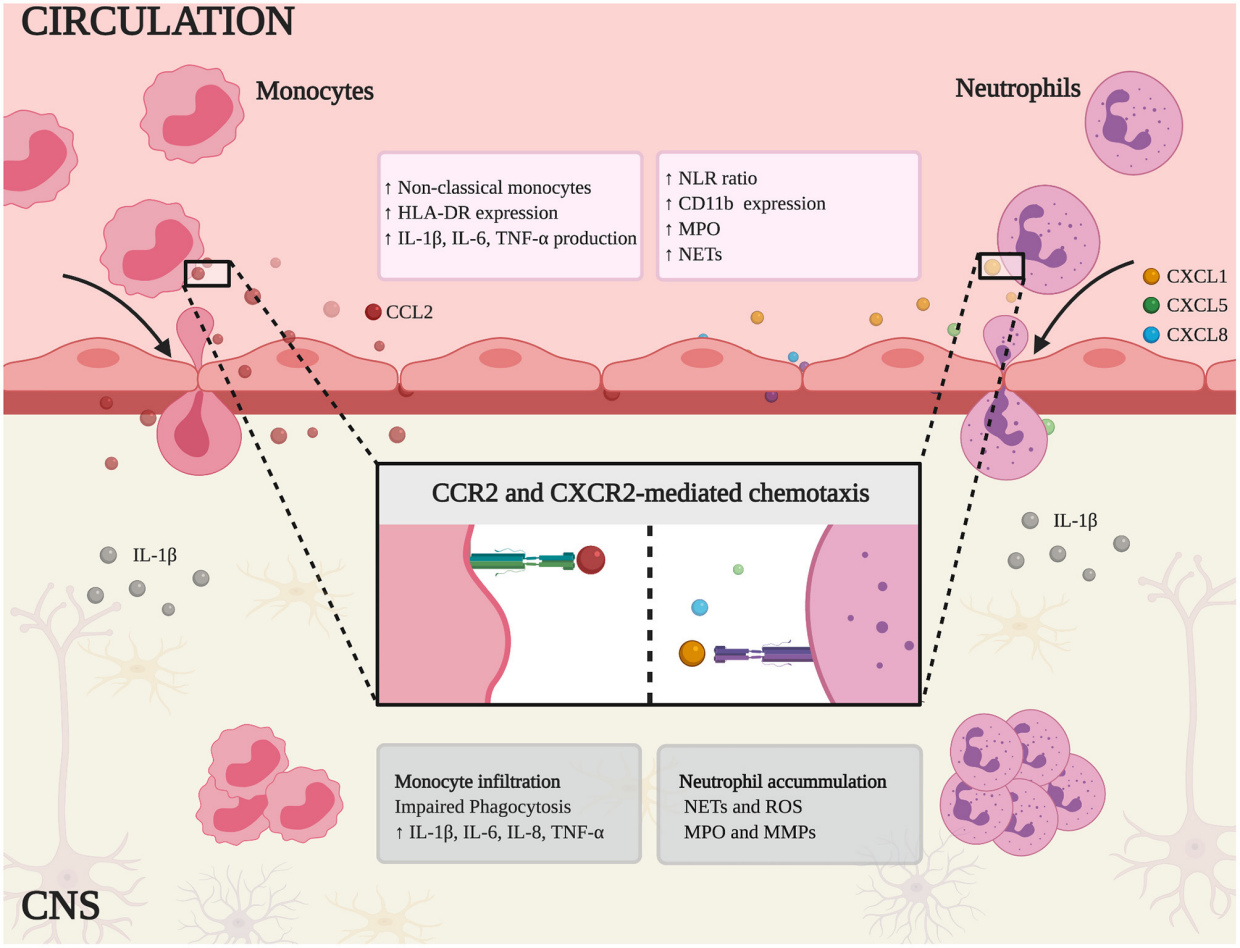
Schematic representation of innate immune mechanisms in MS and AD. Non-classical monocytes and neutrophils are expanded in peripheral blood and the higher neutrophil-lymphocyte ratio (NLR) correlates with the clinical symptoms of each disease. In addition, these cells express canonical activation markers (HLA-DR and CD11b) and release inflammatory mediators, such as pro-inflammatory cytokines (IL-1β, IL-6, TNF-α), myeloperoxidase (MPO), and neutrophil extracellular traps (NETs). Chemokine binding stimulates monocytes and neutrophils to infiltrate the CNS by chemotaxis (mediated by CCR2 and CXCR2, respectively). Within the CNS, monocytes show impaired phagocytosis and secrete the pro-inflammatory cytokines IL-1β, TNF-α, IL-6, and IL-8, thus fueling the inflammatory response. Similarly, infiltrating neutrophils contribute to neuroinflammation and tissue damage by releasing NETs, reactive oxygen species (ROS), MPO, and matrix metal proteinases (MMPs).

In MS and AD patients, the neutrophil to lymphocyte ratio (NLR) is a classical blood marker of inflammation. In MS, the NLR increases during progression and relapses, whereas in AD it correlates with cognitive impairment (32–35). In both diseases, a large proportion of circulating neutrophils is primed, as shown by the induction of activation markers such as CD11b and CD177 (36–39). Interestingly, high levels of CD11b also coincide with relapses in MS patients and correlate with the severity of the cognitive deficit in AD, suggesting that circulating neutrophils with a primed phenotype may cross the cerebral vasculature to the CNS more readily (36, 37). A similar mechanism has been proposed for CD11a/CD18 (LFA-1 or α_L_β_2_) in the recruitment of neutrophils in AD models (10). Furthermore, peripheral hyper-activated neutrophils secrete inflammatory mediators and intravascular neutrophil extracellular traps (NETs), thus contributing to BBB damage and disease development (10, 24, 40) (Figure 1, Table 1).

**Table 1:** Summary of common pathways in the innate immune system during the development of MS and AD.

**Common mechanisms**	**MS**	**AD**
CNS neutrophil infiltration is related to disease progression	(26) (human) (27) (human) (28) (mouse) (29) (mouse)	(10) (mouse)
High NLR correlates with disease progression	(33) (human) (34) (human)	(32) (human) (35) (human)
Circulating neutrophils display a primed-activated phenotype	(36) (human) (38) (human) (40) (human)	(37) (human) (39) (human) (41) (human) (42) (human) (43) (mouse)
CD11b expression on circulating neutrophils correlates with disease progression	(36) (human)	(37) (human)
CXCL8 is elevated in the plasma and CSF and is related to disease activity	(44) (human) (45) (human)	(46) (human) (47) (human) (48) (human)
Elevated CXCL1 expression in the CNS is related to clinical impairment	(36) (human) (44) (human) (49) (mouse)	(50) (human) (51) (mouse)
Elevated IL-1 expression in the CNS	(52) (human) (53) (mouse)	(54) (human) (55) (mouse)
Increased levels of MPO in the blood and CNS correlates with neuropathology	(56) (human) (57) (human) (58) (mouse) (59) (mouse)	(46) (human) (60) (human) (10) (human and mouse)
Circulating neutrophils show a more intense oxidative burst	(40) (human)	(41) (human) (42) (human)
Systemic phenotype alteration in circulating monocytes (increased frequency of non-classical monocytes at the expense of classical ones)	(61) (human) (62) (human)	(63) (human)
Circulating monocytes display a pro-inflammatory state	(64) (human)	(63) (human) (65) (human and mouse)
CCR2 is involved in monocyte CNS invasion	(66) (mouse)	(67) (mouse) (68) (mouse)
Monocytes display impaired phagocytosis and an enhanced pro-inflammatory phenotype	(69) (human)	(70) (human)

During inflammation, the constitutive expression of CXCR2 (a chemokine receptor for the ELR^+^ chemokines CXCL1-3 and CXCL5-8) on mature neutrophils is strongly associated with neutrophil mobilization from the bone marrow to the bloodstream and their migration from the bloodstream to the site of injury (71). CXCL8, a CXCR2-dependent neutrophil chemoattractant, is more abundant in the plasma and CSF of MS and AD patients, and is linked to disease activity, suggesting that neutrophil migration is relevant in both diseases (44–48). Moreover, activated astrocytes produce CXCL1 (another CXCR2 ligand) at the lesion edges in EAE mice, and high levels of CXCL1, CXCL5, and CXCL8 are detected in the serum of MS patients, supporting a role for CXCR2 in the infiltration of neutrophils into the CNS in this disease (36, 44). CXCL1 is also produced by oligodendrocytes in EAE mice, attracting neutrophils into the CNS, exacerbating clinical impairment and enhancing BBB leakage (49). Interestingly, in the CNS of EAE mice, infiltrating T helper (Th) 17 cells stimulate the local release of CXCL1 and CXCL2, which leads to neutrophil recruitment (72). On the other hand, microglia in murine models of AD express CXCL1, and the levels of this chemokine in the CSF of AD patients correlate with cognitive impairment, suggesting that CXCL1 is also important in AD (50, 51) (Figure 1, Table 1). Restricting the infiltration of neutrophils using inhibitors of CXCR1 and CXCR2 has shown therapeutic efficacy in several experimental models of neuroinflammation including EAE, suggesting this may also be the case in animals with AD-like disease (73–75).

Amyloid β may also play a role in both AD and MS (76, 77). In AD brains, infiltrating neutrophils are closely associated with amyloid β deposits, and amyloid β peptides trigger the rapid integrin-dependent adhesion of neutrophils via G protein coupled receptors (10, 78). The non-random distribution of myeloperoxidase (MPO)-producing cells, presumably neutrophils, in the brain parenchyma of AD patients underlines the potential role of amyloid β as a chemoattractant that establishes a pro-inflammatory microenvironment to recruit circulating neutrophils (10). We speculate that the presence of amyloid β deposits in MS could also help to recruit neutrophils into the brain. The abundance of MPO and elastase in the blood and CNS of MS patients suggests that neutrophils may contribute to these pathological findings (56, 57). Moreover, neutrophil elastase is associated with the spread of MS lesions and clinical progression, whereas peripheral MPO activity is considered a predictor for executive function decline in AD patients (29, 46).

Neutrophil migration into the CNS during early or late phases of neuroinflammation plays a crucial role in BBB impairment. During migration, “outside-in” signaling generated following selectin and integrin engagement can induce ROS production by direct NADPH oxidase complex activation and release of other inflammation mediators such as cytokines (79–83). Moreover, it has been previously shown that intravascular neutrophil adhesion *per se* induces alterations in vascular permeability supporting a role for these cells in BBB breakdown (84–86). The inhibition of MPO and elastase in EAE mice reduced the number of infiltrating neutrophils, restored the integrity of the BBB, and attenuated the clinical symptoms (58, 59). MPO-producing cells were also identified in the brain parenchyma of AD patients and corresponding animal models (10). MPO and elastase are involved in the production of NETs, whose release in the CNS correlates with neuronal damage and BBB breakdown (57, 87). Indeed, the formation of NETs occurs in both MS and AD, strongly suggesting a role for neutrophils in the brain damage associated with both diseases (10, 40). Moreover, circulating neutrophils from MS and AD patients display a stronger oxidative burst, which may contribute to the formation of NETs, the activation of matrix metalloproteinases (MMPs), and therefore to BBB breakdown (40–42) (Figure 1, Table 1).

Taken together, these data suggest that activated circulating neutrophils mediate BBB damage and neurotoxicity in MS and AD by producing inflammatory mediators such as MPO and ROS, and by releasing NETs (88, 89). The blocking or inhibition of neutrophil activity could therefore achieve therapeutic benefits for both MS and AD patients (Figure 1).

## The Role of Heterogeneous Monocytes in MS and AD

Circulating monocytes are heterogeneous and plastic innate immune cells that can promptly respond to changes in their environment. Traditionally, human monocytes are divided into three subsets: (i) classical (CD14^+^/CD16^−^), (ii) intermediate (CD14^+^/CD16^+^), and (iii) non-classical (CD14^lo^/CD16^+^). In mice, only two subclasses of monocytes have been identified: (i) classical (CCR2^+^/CX3CR1^−^/Ly6C^hi^), and (ii) non-classical (CCR2^−^/CX3CR1^+^/Ly6C^lo^) (90). In addition to the differential expression of surface markers, these subsets show transcriptional and functional differences: classical monocytes are the main subset recruited to sites of infection and injury (91), whereas non-classical monocytes circulate in the blood, patrolling the vasculature (92).

Systemic alterations in monocyte subsets have been reported in humans with neurodegenerative disorders. The dysregulation of monocyte subsets in patients with RRMS involves the expansion of non-classical and intermediate monocytes and the depletion of classical monocytes (61, 62). Similarly, AD patients with symptoms ranging from very mild to severe dementia accumulate non-classical and intermediate monocytes at the expense of classical monocytes (63) (Figure 1, Table 1). Although changes in circulating monocyte subsets have been confirmed across diverse CNS diseases, the meaning, causes and consequences of these alterations are still unclear. Whether different monocyte subsets correspond to developmental stages or whether each monocyte subset has a different developmental pathway remains to be determined. Given the plasticity of monocytes and their ability to respond rapidly to a wide variety of stimuli, the analysis of monocyte activation, and cytokine profiles should indicate their function and contribution to peripheral inflammation during neurodegeneration. Indeed, circulating monocytes in MS and AD patients shift toward a pro-inflammatory phenotype (63, 64) (Figure 1, Table 1). Particularly, in MS patients, isolated monocytes were shown to produce more IL-1β, TNF-α, IL-6, and IL-8 under basal conditions (61). Similarly, unstimulated monocytes from patients with dementia express higher levels of *IL-6, IL-1*β, and *TNF-*α mRNA (63) (Figure 1), suggesting a pro*-*inflammatory phenotype in AD. Interestingly, *IL-8*, and *TNF-*α mRNA levels also increase when human monocyte-like cell line THP-1 is incubated with plasma from AD patients or transgenic mice with AD-like disease, compared to plasma from healthy human controls or wild-type mice, respectively (65). This provides evidence that systemic inflammatory conditions affect the function of circulating monocytes.

Monocytes can migrate into tissues and differentiate into macrophages, making them important players in brain homeostasis. However, the correct identification of these cells in the CNS has proved challenging due to the similarities between microglia and monocyte-derived macrophages, especially during inflammation when the surface markers change. This has been addressed by advances in genome editing and cell tracing technology, leading to the identification of CCR2 and CX3CR1 as markers of murine classical monocytes and microglia, respectively (93). In this context, establishing how circulating monocytes are recruited to the inflamed brain can contribute significantly to the understanding of pathophysiology in MS and AD. Studies in murine models revealed the essential role of CCR2, a chemokine receptor involved in mononuclear trafficking at inflammation sites (94). In the EAE model of MS, classical monocytes were found to infiltrate the inflamed brain and the ablation of CCR2 blocked this process, suggesting a key role for this receptor in the recruitment of monocytes to the CNS during EAE (66). The same phenomenon has been observed in AD mice, where the loss of CCR2 reduces the number of monocytic phagocytes in the brain (67, 68) (Figure 1, Table 2). Together, these findings suggest that classical monocytes are the main subset that invades the CNS in neuroinflammatory conditions such as MS and AD and that CCR2 plays a fundamental role in the recruitment of these cells into the CNS. Interestingly, while CCR2 blockade in EAE has a beneficial effect, inhibition of this receptor in AD models increases Aβ deposition and worsens memory deficits, suggesting a decreased expression of CCR2 could play a potential role in the etiology of AD. Accordingly, the number of monocytes is lower in AD mouse models than controls, mostly due to the depletion of CCR2^+^ monocytes, suggesting these cells are severely impaired in AD (140). Also, in AD patients, CCR2 expression decreases in circulating monocytes whereas the levels of plasma CCL2 were increased, suggesting systemic immunologic dysfunction CCR2-CCL2 axis (141, 142) (Figure 1). Although mouse models of MS confirm the close involvement of monocytes in disease pathogenesis, clinical trials using CCR2 antagonists did not demonstrate efficacy (EU Clinical Trials: https://www.clinicaltrialsregister.eu/ctr-search/search?query=2004-000073-64). Indeed, CCR2^+^ monocytes can be immunosuppressive, they replenish important macrophage populations, and they play pivotal roles during infection, potentially explaining the lack of positive results following a CCR2 therapeutic blockade in MS (68, 143, 144).

**Table 2:** Summary of common pathways in the adaptive immune system during the development of MS and AD.

**Common mechanisms**	**MS**	**AD**
CD4^+^ cells infiltrate CNS	(95) (human) (96) (human) (97) (human)	(98) (human) (99) (human) (100) (human)
Increased frequency of circulating Th17 cells and serum level of IL-17	(101) (human) (102) (human)	(103) (human) (104) (human)
α4-integrin is involved in CNS invasion by CD4^+^ T cells	(105) (mouse) (106) (human)	(31) (mouse) (107) (mouse)
CD4^+^ T cells interact with microglia expressing MHC-II at high levels	(108) (human) (109) (rat)	(110) (human) (111) (mouse)
CD4^+^ T cells work along the gut–brain axis to modify cognitive functions	(112) (mouse)	(113) (mouse) (114) (mouse and human)
CD8^+^ T cells infiltrate the CNS and trigger detrimental effects	(115) (human) (116) (human)	(100) (human) (117) (human) (98) (human) (118) (mouse)
Increased proportion of circulating CD8^+^ T cells	(119) (human) (120) (human)	(121) (human)
Circulating CD8^+^ T cells show a primed-activated phenotype	(122) (human) (123) (human) (124) (human)	(117) (human) (125) (human)
Clonally expanded CD 8^+^ T_EMRA_ cells	(126) (human)	(117) (human)
CD 8^+^ T cells clonally respond against EBV	(127) (human)	(117) (human)
Lower number of circulating CD4^+^ CD25^+^ FoxP3^+^ cells	(128) (human) (129) (human)	(130) (human)
T_reg_ cells with impaired regulatory activity	(131) (human) (132) (human)	(133) (human) (134) (human)
Depletion of T_reg_ cells associated with worst outcomes	(135) (mouse) (136) (mouse) (137) (mouse)	(138) (mouse) (139) (mouse)

Infiltrating monocytes and resident microglial cells can both react to inflammatory stimuli and mount an immune response in the CNS during neurodegenerative diseases (144). Indeed, monocytes not only infiltrate the CNS parenchyma but also colonize the meninges in EAE mice (145). Furthermore, monocyte infiltration begins at the onset of the disease and continues to increase until the disease peak, suggesting a role for these cells in disease induction and progression (145). Moreover, monocyte-derived cells infiltrating the CNS are major players in antigen presentation during EAE and recent studies identified several subtypes of infiltrating monocytes/myeloid cells in the CNS with different transcriptional landscapes during the acute and chronic stages of EAE (146). Similarly, single-cell studies revealed disease-specific transformations across several types of brain-associated phagocytes in murine models of AD, but the existence of common signatures between EAE/MS and AD is unclear (147). In AD mice, circulating monocytes have been shown to invade the brain and reduce amyloid β burden, suggesting a beneficial role for these cells in AD (148). Also, patrolling monocytes have been described to crawl onto the luminal walls of amyloid β-positive veins, suggesting their ability to target and clear amyloid β (149). The same applies to perivascular macrophages in another mouse model of AD, in which their depletion led to an increased accumulation of amyloid β deposits in blood vessels (150). Brain macrophages are not only involved in the clearance of CNS debris or amyloid β, but also play an important role in regulating iron levels. Extracellular accumulation of iron during neurodegeneration can be attributed to an array of processes including oligodendrocyte and myelin degeneration (151, 152). Indeed, increased iron deposits in white matter lesions have been shown in both MS and AD, and iron accumulation correlates with cognitive deficits (153, 154). Interestingly, the deposition of iron observed in MS was often co-localized with microglia/macrophages, which express the transferrin receptor, a main iron influx protein. By capturing iron and, therefore, preventing Fenton reactions and the creation of oxygen radicals, macrophages play an important regulatory function in the inflamed brain during MS (155, 156). In AD, however, there is limited information on iron uptake by macrophages, although recent evidence suggests that stimulation of microglia with Aβ increases the uptake of non-bound iron by these cells (157).

However, although monocytes infiltrate the brain and, to some degree, remove debris, iron and amyloid β, these cells in mice with AD-like disease are ineffective in clearing amyloid β in the diseased brain and their peripheral phenotype changes to a pro-inflammatory profile with limited phagocytic ability. Indeed, peripheral blood monocytes from AD patients cannot differentiate normally *in vitro* and have a lower capacity for the uptake of amyloid β uptake, eventually leading to apoptosis (70). Similarly, circulating monocytes in MS patients also adopt a pro-inflammatory profile with limited phagocytic ability, thus failing to promote remyelination and repair through the removal of myelin debris by phagocytosis (69) (Figure 1, Table 1). Collectively, these data suggest that chronic systemic inflammation in MS and AD leads to common pathological changes among the population of circulating monocytes. Understanding the role of these cells may provide insight into the disease mechanisms and lead to new therapeutic targets in neurodegenerative disorders.

## The Role of T Cells in the Development of MS and AD

T lymphocytes are cells of the adaptive immune system that provide specific responses to eradicate pathogens or antigens that act as elicitors (158). Depending on their function, T lymphocytes can be subdivided into three main classes: (i) CD8^+^ cytotoxic T (Tc) lymphocytes responsible for the elimination of infected somatic cells, (ii) CD4^+^ T helper (Th) lymphocytes that assist and guide other immune cells, and (iii) regulatory T (T_reg_) lymphocytes associated with the attenuation and resolution of inflammation. The pathological dysregulation of the adaptive immune system promotes chronic and uncontrolled inflammatory reactions that may eventually lead to tissue damage. MS and AD are both characterized by a chronic neuroinflammatory pathology (159, 160). In MS, T cells are known to play an essential role in disease pathogenesis but the extent to which T cells contribute to the pathology of AD is less clear. In MS, T cells react against myelin autoantigens, migrate across a damaged BBB, accumulate in active lesions, and trigger damage to myelin and underlying axons, thus promoting all classical MS symptoms (161). Similarly, the post-mortem analysis of brains from AD patients revealed the accumulation of brain-infiltrating T cells, and recent experimental evidence from animal models of AD suggest a pathological role for these cells (100, 117). In support of this, the sequestration of T lymphocytes in lymphoid organs induced by fingolimod treatment decreases the number of circulating T cells and ameliorates disease in MS patients and animal models of AD (162–166).

### CD4^+^ T Lymphocytes in MS and AD

CD4^+^ T cells infiltrate both the white and gray matter of MS patients, and similar observations have been reported in AD brains (95–100) (Table 2). Pathogenic CD4^+^ T cells in MS and EAE have been subtyped by cytokine profiling, revealing Th1 cells that produce IFN-γ and Th17 cells that produce IL-17. Both Th1 and Th17 cells are key components of the autoimmune inflammatory process during the development of MS, and they may fulfill analogous roles in AD. Following CNS invasion, Th1 and Th17 cells produce inflammatory mediators and cytokines to establish and/or maintain an inflammatory environment that promotes neuronal loss, a common feature of MS and AD that positively correlates with the disease course (167, 168). In particular, the infiltration of Th17 cells into the CNS of MS patients increases the concentration of IL-17 in the blood and CSF and the number of Th17 cells found in these compartments (101, 102, 169). Similarly, IL-17 also accumulates to higher levels in the serum of AD patients compared to healthy controls and has been proposed as part of a blood-based signature to distinguish individuals with AD from healthy subjects (104, 170). The population of circulating Th17 cells has also been shown to increase in MCI patients compared to both age-matched controls and AD patients, suggesting that Th17 cells may be involved in the early stages of AD (103) (Table 2). Intriguingly, EAE mice modified to abolish IL-17 production, as well as AD models treated with neutralizing antibodies against IL-17, show delayed clinical progression, confirming the harmful effects of IL-17 in EAE and suggesting that Th17 cells also contribute to the progression of AD (171).

**Figure 2:**
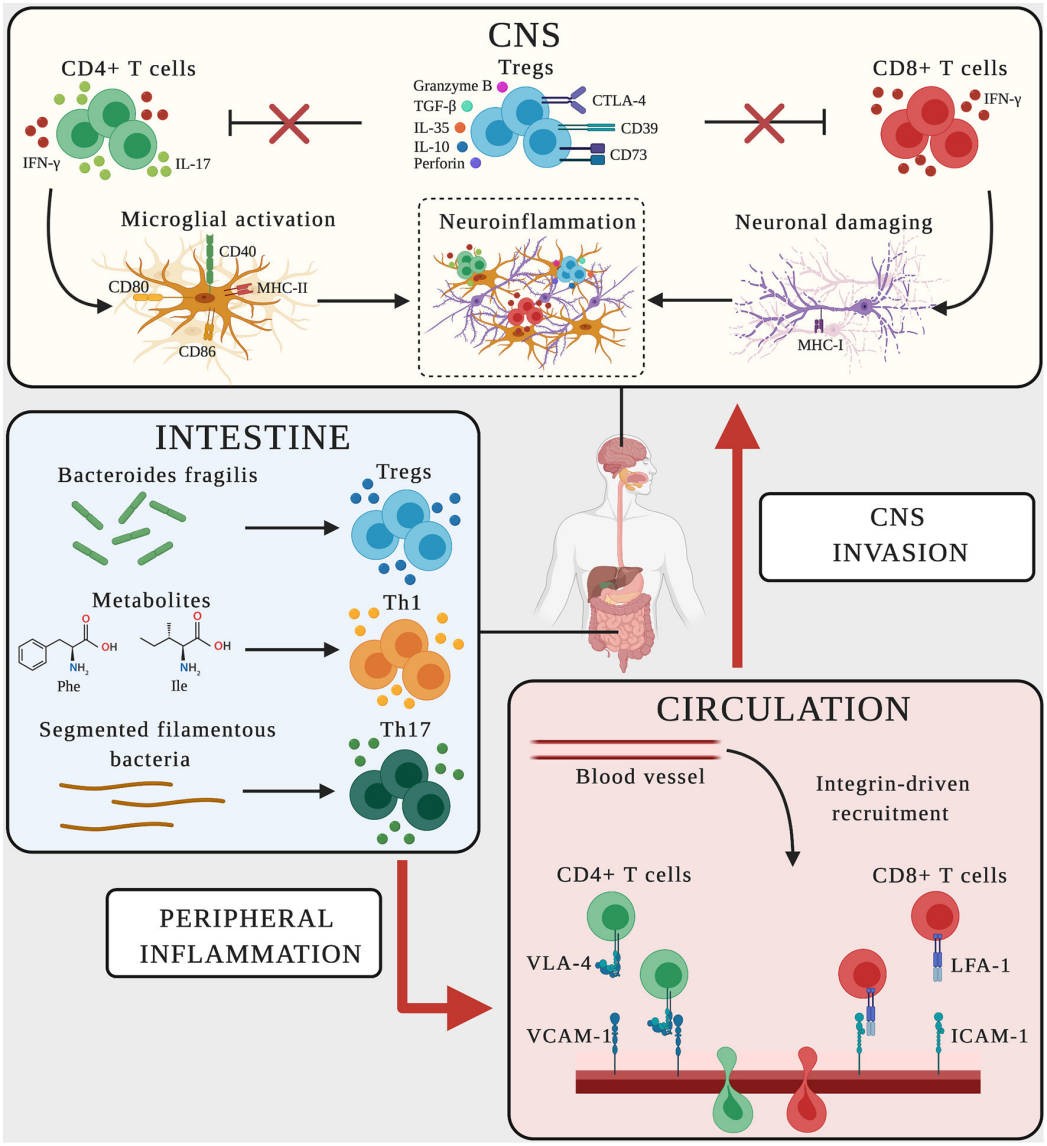
Schematic representation of adaptive immune mechanisms in MS and AD. In CNS, CD4^+^ T cells producing the pro-inflammatory mediators IFN-γ-and IL-17 promote microglial activation, upregulating the expression of CD40, MHC-II, CD80, and CD86 on the surface and favoring neuroinflammation. CD8^+^ T cells producing IFN-γ bind neurons expressing MHC-I, triggering neuronal damage and boosting neuroinflammation. Dysregulated T_reg_ cells fail to suppress effector T cell functions (red crosses), thus sustaining the neuroinflammatory environment. In the intestine, the microbiome and its metabolites influence the polarization and activation of T cells. *Bacteroides fragilis* promotes the expansion of T_reg_ cells, the amino acids phenylalanine and isoleucine induce the differentiation of Th1 cells, and segmented filamentous bacteria trigger Th17 cell polarization. Vascular endothelial cells express the LFA-1 and VLA-4 counter-ligands (ICAM-1 and VCAM-1) guiding the transmigration of peripherally activated T cells from the circulation to the CNS.

CD4^+^ T cells appear to invade the CNS of AD and MS patients using common molecular pathways, emphasizing the common leukocyte recruitment mechanisms in these two diseases. For example, several studies in MS/EAE have shown that α4-integrins play a key role in the trafficking of Th cells (105, 106). EAE progression is delayed following the selective deletion of α4-integrin genes in T cells and MS progression is delayed by treatment with the α4-integrin-blocking humanized antibody natalizumab (172, 173). We and others recently demonstrated a similar molecular mechanism controlling the infiltration of Th cells in AD models (31, 107). We observed the stronger expression of α4-integrins on circulating CD4^+^ T cells in an AD mouse model compared to age-matched controls, along with an increase in the abundance of CD4^+^ cells in the brains of AD mice (31) (Figure 2, Table 2). Importantly, blocking α4-integrins inhibited the adhesion of circulating leukocytes in the brain microcirculation and reduced the neuropathological hallmarks of AD, highlighting the potential for a therapeutic approach that is similar in efficacy to the use of natalizumab in MS patients (172, 173).

In EAE models, lymphocytes are presented with antigens in the periphery before CNS invasion. Indeed, T-cell priming begins in secondary lymphoid organs and leads to the activation and expansion of neuroantigen-reactive T cells that later infiltrate the CNS, where they re-encounter their cognate antigen (174). Within the CNS, microglial cells may promote the proliferation and activation of CNS-reactive T lymphocytes (174, 175). In both MS and AD, activated microglia express the main major histocompatibility complex class II molecule (MHC-II) as well as co-stimulatory molecules such as CD40, CD80, and CD86, which equip the microglia for antigen presentation to infiltrating T cells, creating a vicious cycle that promotes neuroinflammation and potentially antigen presentation (108–111) (Figure 2, Table 2). As a result, the pro-inflammatory environment that can activate CD4^+^ T cells is continuously boosted, and may promote neuronal damage in both MS and AD (Figure 2).

Although CD4^+^ T cells are considered pathogenic in several CNS disorders, they may also provide beneficial functions in AD, ranging from tissue protection to regeneration (176). In AD models, amyloid β-reactive T cells effectively target amyloid β plaques in the brain, enhancing phagocytosis by microglia and leading to neuronal repair (177). Furthermore, Th1 cells injected into the ventricles of AD mice were able to induce the differentiation of microglia (protective MHC-II^+^ subtype), boosting the capacity for amyloid β clearance (111). Despite these tantalizing results, a protective role for CD4^+^ T cells has yet to be confirmed in AD, and immunotherapeutic approaches based on amyloid β have not achieved efficacy in clinical trials (178).

### T_reg_ Cells and Their Failure to Control Inflammation in MS and AD

T_reg_ cells fulfill an active regulatory role in peripheral tolerance mechanisms, preventing the onset of autoimmunity and limiting chronic inflammation. They downregulate the activities of various immune cells, including effector T cell functions and proliferation, by the secretion of immunosuppressive cytokines (including TGF-β, IL-10 and IL-35) and/or by direct cytotoxicity and the induction of apoptosis (through the release of granzyme B and perforin 1) (179–181). T_reg_ cells also cause indirect immunosuppression via cytotoxic T lymphocyte antigen 4 (CTLA4), CD39, and CD73, and disrupt the metabolism of the effector T cells by modulating the maturation and/or function of the dendritic cells (DCs) required for their activation (182).

Dysfunctional T_reg_ cells have been linked to neuroinflammatory conditions, and the analysis of peripheral blood demonstrates how T_reg_ cells can contribute during neurodegenerative diseases. T_reg_ cells have recently been shown to infiltrate the brain and suppress astrogliosis by producing amphiregulin in a model of ischemic stroke, but their role during AD and MS is unclear (183). Several studies have shown that the number of circulating T_reg_ cells declines in both MS and AD patients compared to matched controls, suggesting that their dysregulation in the periphery reduces their immunosuppressive capacity and promotes uncontrolled inflammation (128–130). Indeed, T_reg_ cells isolated from the peripheral blood of MS patients show an impaired ability to modulate CD4^+^ T cell proliferation and IFN-γ production (131). Compared to healthy controls, RRMS patients also produce more Th1-like Foxp3^+^ T cells that secrete IFN-γ and show a limited reduced immunosuppressive capacity (132). Similarly, the immunosuppressive functions of T_reg_ cells in AD patients are compromised compared to both healthy controls and subjects with MCI (133, 134) (Table 2). These data are supported by animal models discussed below, where T_reg_ cells appear to be important in both EAE and AD, especially during the early phases of both diseases, but at later stages they are depleted and/or dysfunctional and are therefore unable to control the inflammatory response.

The depletion of T_reg_ cells by anti-CD25 antibodies in EAE mice increased the severity of the disease, boosting the production of IL-17 and T cell infiltration (135). In line with the protective role of T_reg_ cells, the transplantation of neural stem cells in EAE mice induces remyelination by expanding the T_reg_ cell population (136, 137). Similarly, the depletion of T_reg_ cells in mice with AD-like disease is associated with the premature loss of cognitive functions and a worse outcome (138). Furthermore, low doses of IL-2 increased the number of T_reg_ cells in the peripheral blood and lymphoid organs of these mice, restoring their cognitive ability (138). Similar results were reported by others, showing that T_reg_ cell depletion for 4 months during the early stages of AD-like disease aggravated the cognitive deficits and increased the deposition of amyloid β plaques (139). In these studies, the adoptive transfer of purified T_reg_ cells improved cognitive functions and reduced the amyloid β burden (139) (Table 2). Curiously, T_reg_ cell depletion during the late disease stage in an aggressive model of amyloidosis also conferred a beneficial effect, presumably by boosting the immune response, suggesting that the effect of T_reg_ cells on AD-like disease is stage-dependent (184). Taken together, these results suggest that T_reg_ cells play a key role in controlling the development of neuroinflammation in both MS and AD (Figure 2). Therapeutic strategies aiming to exploit the immunosuppressive properties of T_reg_ cells may therefore help to address both pathologies.

### CD8^+^ T Lymphocytes in AD and MS

CD8^+^ T cells appear less heterogeneous than CD4^+^ T cells, but the functional classification of this population is not completely clear. Nevertheless, cytotoxic lymphocytes play a prominent role in the development of many viral and non-viral diseases by the direct killing of infected or otherwise modified cells. Following antigen recognition, cytotoxic T cells classically induce apoptosis in target cells via two alternative mechanisms: (i) FasL-CD95 (FasR) binding to activate caspase, and (ii) the release of granzyme B and perforin. CD8^+^ effector T cells may also cause cellular damage indirectly by secreting the pro-inflammatory cytokines TNF-α and IFN-γ (185). During immune responses, CD8^+^ T cells interact with CD4^+^ T cells to optimize the precision of CD8^+^ T cell effector functions after priming by enhancing their cytotoxicity and capacity for migration (186).

CD8^+^ T cells have received less attention than CD4^+^ T cells in MS and EAE, but it is now well-established that CD8^+^ T cells are more activated in the periphery and can infiltrate active lesions, contributing to the increasing severity of MS symptoms (115, 116, 187). Furthermore, clonally expanded cytotoxic T cells exacerbate brain inflammation in EAE initiated by CD4^+^ T cells, suggesting that CD8^+^ T cells may be primarily responsible for the observed cerebral alterations (187) (Table 2).

The role of CD8^+^ T cells in AD is less clear, although these cells were first detected in the brains of AD patients almost 20 years ago (100). The infiltration of CD8^+^ T cells into the brains of AD patients and corresponding mouse models also correlates with disease worsening, suggesting these cells may be involved in disease development (98, 100, 118). CD8^+^ T cells were shown to accumulate in an active state in the peripheral blood of MS patients and in AD patients with dementia (119–121) (Table 2). The comparison of blood samples from AD patients and healthy controls revealed the production of more pro-inflammatory cytokines by cytotoxic T cells in the AD patients and a greater proportion of activated HLA-DR^+^ CD8^+^ T cells (117, 125). Similarly, circulating CD8^+^ T cells from patients in the acute phase of RRMS showed increased adhesion to brain venules compared to control cells, further highlighting their activated phenotype (122). In line with this, the characterization of CD8^+^ T cells from the CSF and brain tissue of MS patients showed their activated/memory phenotype (123, 124) (Table 2). Notably, a higher frequency of CD8^+^ effector memory T cells was detected in the CSF of AD patients with dementia compared to controls, suggesting CD8^+^ T cells contribute to brain damage in both AD and MS with similar underlying mechanisms (117, 121).

The extravasation of cytotoxic T cells in the brain promotes the brain damage caused by CD8^+^ T cells and appears to be mediated by LFA-1 integrin (CD11a/CD18) in both MS and AD (Figure 2). LFA-1 is a marker of leukocyte activation that binds ICAM-1, which is overexpressed by endothelial cells in several neuroinflammatory conditions (10, 188). CD11a expression was shown to increase on clonally expanded CD8^+^ T cells in MS patients, promoting their transmigration into the brain (116). Without referring to specific T cell populations, previous studies have shown that the transfer of encephalitogenic CD11a^−/−^ T cells to wild-type mice reduces the severity of EAE, suggesting that LFA-1 also facilitates the migration of CD8^+^ T cells in this disease (189). The presence of LFA-1^+^ T cells infiltrating the hippocampus of AD patients suggests a role for LFA-1 also in AD (100). This is consistent with the increase in *Itgb2* (LFA-1) mRNA levels in the hippocampus of a mouse model of tauopathy (190). Finally, the strong upregulation of ICAM-1 was observed in cortical and hippocampal brain regions invaded by CD3^+^ T cells in mouse models of AD, suggesting that LFA-1 also promotes T cell migration into the AD brain (10, 191). Further studies are needed to confirm that LFA-1 is required for the trafficking of CD8^+^ T cells in AD, but given that LFA-1 is required for cytotoxic T cell activation, maturation, immuno-synapse stabilization and functioning, this integrin is likely to play a key role in driving CD8^+^ responses in AD (192).

Another important topic in the context of MS and AD is the antigenic specificity and clonal origin of CNS-infiltrating CD8^+^ T cells. The analysis of lymphocytes collected from the blood, CSF and brain lesions of many MS patients has shown that CD8^+^ T cells undergo clonal expansion, suggesting that they are activated by specific antigens (115, 123). Other studies in MS patients have identified a population of CD8^+^ T cells specific for myelin proteins, presenting an activated/memory phenotype due to *in situ* antigen presentation (96, 119, 120, 193). Tau protein may fulfill a similar role in AD, representing the potential link between cytotoxic T cells and disease development. Indeed, CD8^+^ T cells accumulate in the hippocampal regions of a mouse model of tau pathology, apparently via a tau-driven transmigration mechanism (190). A correlation between tau pathology and the infiltration of CD3^+^ T cells was revealed by the immunohistochemical analysis of post-mortem AD brains, further supporting the potential association between tau and CD8^+^ T cell-dependent pathology in AD (194).

Clonally expanded CD8^+^ T_EMRA_ cells were recently identified in the CSF of AD patients, suggesting that antigen-experienced cytotoxic cells patrol the intrathecal space of AD patients. Interestingly, as already shown in MS patients, CD8^+^ T cells in the CSF were expanded predominantly against Epstein-Barr viral antigens, suggesting a new link between EBV, CD8^+^ T cells and AD (117, 127). CD8^+^ T_EMRA_ cells accumulated not only in the CSF of AD patients, but also in the blood of RRMS and SPMS patients, suggesting these cells contribute to the progression of both AD and MS by promoting chronic inflammation (126) (Table 2). More detailed studies are required to determine precisely how this cell population fits into the complex pathogenesis of neurodegenerative disorders.

The pro-inflammatory nature of CD8^+^ T_EMRA_ cells is strictly associated with IFN-γ secretion, which occurs mainly during active proliferation (195). One of the harmful functions of IFN-γ is the ability to favor neuronal killing by CD8^+^ T cells via the FasR-FasL pathway (185). Accordingly, previous studies have shown that the overexpression of MHC-I on neurons exposed to IFN-γ promotes neuronal damage by CD8^+^ T cells via TCR-MHC-I binding (196). Interestingly, MHC-I has been detected on neurons in both hippocampal and cortical brain regions, which are heavily damaged in AD (196). Similarly, CD8^+^ T cells in chronic and active MS plaques were found marginally in contact with oligodendrocytes, astrocytes and neurons expressing MHC-I at high levels (96, 96, 193). These results indicate that CD8^+^ T cells producing IFN-γ enhance the expression of MHC-I on neurons in MS and AD, and could therefore promote the brain alterations associated with the progression of both diseases (Figure 2). In conclusion, CD8^+^ T cells appear to drive the development of both MS and AD by sustaining chronic inflammation and directly causing CNS injury.

### The Gut–Lymphocyte–Brain Axis in AD and MS

Several clinical and preclinical studies have shown that the course of MS and AD is influenced by the commensal gut microbiome, highlighting the interplay between the brain, gut microbes, intestinal barrier and immune system (197, 198). Indeed, fecal and mucosa-associated gastrointestinal tract microbes differ between AD patients and healthy controls, and recent studies comparing germ-free animals and those exposed to pathogenic bacteria, probiotics or antibiotics suggested a role for gut microbes in host cognitive functions and the development of AD-like neuropathological features (113, 199–202). Many studies have also focused on the role of gut microbes in MS and EAE (112, 203–208). These studies suggest that altering the gut microbiome with antibiotic cocktails or probiotics can attenuate the disease course by modulating regulatory immune responses (198). Although many of the microbes and metabolites in the gut–CNS axis have been identified, little is known about the underlying cellular and molecular immune mechanisms. In MS, the balance between pro-inflammatory myelin-reactive effector cells and anti-inflammatory immune elements controlling the formation of CNS lesions is continuously influenced by the gut. Indeed, both monocytes and gut-resident macrophages influence the gut-dependent activation of CD4^+^ T cells, which promotes Th1 polarization and IFN-γ secretion (209). Gut microbes and their metabolites also regulate T cell-mediated adaptive immune responses. For example, specific bacteria such as *Akkermansia muciniphila* and *Acinetobacter calcoaceticus* are associated with MS, inducing a pro-inflammatory T cell phenotype that perpetuates autoimmune responses, whereas segmented filamentous bacteria, symbiotic colonizers of the small intestine, induce Th17 differentiation and activation to promote the development of EAE (210, 211) (Figure 2). In contrast, CNS demyelination and inflammation during EAE is inhibited by gut flora rich in the commensal bacterium *Bacteroides fragilis*, which promotes the expansion of T_reg_ cells expressing the ectonucleotidase CD39 and their migration into the CNS (212) (Figure 2). A recent innovative study in SPMS patients confirmed a role for the gut–brain axis in MS patients, showing the depletion of a subset of circulating memory CD4^+^ T cells expressing the gut-homing chemokine receptor CCR9 and the α4β7 adhesion molecule and a tendency to switch from a regulatory to a pro-inflammatory phenotype that produces more IFN-γ and IL-17 (213). Recently, gut dysbiosis in MCI patients was shown to increase phenylalanine and isoleucine levels, correlating with an increase in the number of circulating Th1 cells (114) (Figure 2). Interestingly, naive CD4^+^ T cells exposed to phenylalanine or isoleucine acquire an activated Th1 phenotype, and the oral treatment of mouse models of AD with the prebiotic oligosaccharide GV-971 (which suppresses gut dysbiosis) reduced the concentration of phenylalanine and isoleucine, resulting in the amelioration of neuroinflammation and cognitive impairment (114).

Collectively, these data show that gut dysbiosis contributes to peripheral immune cell dysregulation and triggers an enhanced inflammatory immune response. The gut–brain axis may therefore provide an appropriate target for immunomodulatory therapy in both diseases.

## Conclusions

Neurodegenerative diseases are increasing in prevalence and socioeconomic impact. The identification of common cellular and molecular mechanisms involving the immune system may provide more insight into pathogenesis, leading to potential common therapeutic strategies. Several genome-wide association studies (GWAS) in both MS and AD patients have revealed associations between these diseases and gene expression in peripheral adaptive and innate immune cells, suggesting common immune mechanisms controlling neuroinflammation and neurodegeneration (214–216). Moreover, functional studies *in vitro* and *in vivo* have revealed shared detrimental molecular mechanisms in peripheral leukocytes, representing potential common therapeutic targets for the control of immune responses in MS and AD (217–219). One clear example of such a shared molecular mechanism is represented by α4 integrins, which can be targeted in both MS and AD. Indeed, the therapeutic effect of α4 integrins in EAE and MS has been corroborated by recent studies showing that targeting these adhesion molecules reduces neuroinflammation and the neuropathological hallmarks of AD (31). Moreover, reducing BBB breakdown that characterizes several neurodegenerative disorders, including MS and AD, could be considered as another common possible therapeutic strategy in reducing not only the influx of various neurotoxic agents, but also the recruitment of immune cells into the CNS (220, 221). By using animal models of both MS and AD, it was indeed demonstrated that targeting BBB pathways to preserve vascular integrity, ameliorates the course of brain pathology (222, 223). Therefore, a deeper understanding of the activation status of peripheral innate and adaptive immune cells in the blood, their trafficking mechanisms, CNS pathogenic signatures, and neurotoxic effects, may lead to the discovery of new common biomarkers of MS and AD that could facilitate the identification of common therapeutic strategies for both diseases.

## Author Contributions

All authors contributed to the literature search and writing the review.

## Conflict of Interest

The authors declare that the research was conducted in the absence of any commercial or financial relationships that could be construed as a potential conflict of interest.
